# Evaluation of disinfection byproduct formation from extra- and intra-cellular algal organic matters during chlorination after Fe(vi) oxidation

**DOI:** 10.1039/c9ra06449d

**Published:** 2019-12-12

**Authors:** Feilong Dong, Qiufeng Lin, Cong Li, Tuqiao Zhang

**Affiliations:** School of Environment and Architecture, University of Shanghai for Science and Technology Shanghai 200433 China congil@aliyun.com; College of Civil Engineering and Architecture, Zhejiang University Hangzhou 310027 China

## Abstract

Blue-green algae commonly bloom in fresh water in summer, producing extra- and intra-cellular algal organic matters, which are important precursors for disinfection byproduct (DBP) formation. In this study, we evaluated the effect of pre-oxidation with ferrate(vi) (FeO_4_^2−^, Fe(vi)) on the characterization of intracellular organic matter (IOM) and extracellular organic matter (EOM). The results indicated that soluble microbial-like products of EOM and IOM decreased and humic acid-like products of IOM increased, which would influence the DBP formation in the subsequent chlorination step. Therefore, in this study, the effect of Fe(vi) pre-oxidation on the DBP formation from IOM and EOM with subsequent chlorination was also investigated. For *Chlorella* sp., EOM presented no significant change, and IOM presented a reduction of THMs (8.2%) after Fe(vi) oxidation at a dosage of 16 mg L^−1^. For *P. limnetica*, EOM and IOM both exhibited reduction of trihalomethanes (THMs) and chloral hydrate (CH) after Fe(vi) oxidation. Besides, THMs had the lowest concentration at pH 8.0 in all four solutions. Haloacetonitriles (HANs) and haloketones (HKs) showed slight changes with increasing pH values. Due to the frequent detection of bromide (Br^−^) in surface water, the effect of bromide existence on THM formation was also investigated. The results indicated that all brominated DBPs increased, and chlorinated DBPs decreased with the increase in bromide concentration. In addition, the bromine substitution factor (BSF) of *Chlorella* sp. and *P. limnetica* both increased with the increase in Br^−^ concentration.

## Abstract 2

Blue-green algae commonly bloom in fresh water in summer, producing extra- and intra-cellular algal organic matters, which are important precursors for disinfection byproduct (DBP) formation.

## Graphical abstract

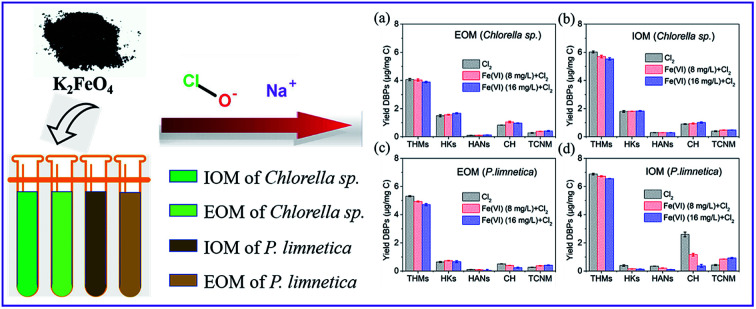

## 1. Introduction

Algal blooms (or blue-green algae, *e.g. Microcystis aeruginosa*, *Chlorella* sp., *P. limnetica*) occur frequently in fresh water bodies due to water eutrophication, which has been a problem worldwide.^1,2^ The increasing algal cells generate algal organic matter (AOM) as a byproduct of cell lysis, certified to generate undesirable matters (*e.g.* taste, odour and algal toxins) and cause a series of problems during water treatment (*e.g.* forming the disinfection byproducts (DBPs)).^3–5^ Trihalomethanes (THMs) and haloacetic acids (HAAs) are the mainly halogenated DBPs derived from AOM during chlorination (accounting for more than 50%), which are currently regulated by the Environmental Protection Agency (EPA).^6–8^ However, some of the more toxic DBPs such as haloketones (HKs), haloacetonitriles (HANs) and halonitromethanes (HNMs), which were proved to be carcinogenic for humans, are also produced during the AOM chlorination process.^9,10^

AOM contains extracellular organic matter (EOM) and intracellular organic matter (IOM), which consist of carbohydrate, amino acids and proteinaceous compounds.^5^ IOM could be released by algal metabolism, excretion and autolysis through the pre-oxidation of ozone, chlorine and Fe(vi).^11,12^ IOM and EOM, which were the main precursors of DBPs, increased with the increase in the age of the algal system.^13^ Due to the difficulty in their removal during regular water treatment (*e.g.* coagulation and sedimentation),^14^ it is of interest to investigate the DBP formation from EOM and IOM by Fe(vi) treatment. Therefore, a series of pre-treatment processes were used to degrade AOM and reduce DBP formation. Zhu *et al.* investigated the effect of pre-ozonation on DBP formation from *Microcystis aeruginosa*.^15^ They found that HANs decreased by 9.4% after ozonation from the chloramination of IOM, but bromate was generated simultaneously. Xie *et al.* found that most nitrogen-DBPs (N-DBPs) increased slightly after per-oxidation with permanganate.^12^ Fe(vi) has been used as an environmentally friendly water treatment oxidant in recent years^16^ because it has strong redox potentials of +2.2 V and +0.72 V in acidic and alkaline solutions, respectively, and decomposed nontoxic Fe(iii) ions as coagulants.^17^ Zhou *et al.* studied the THM and HAA formation after Fe(vi) pre-oxidation from *Microcystis aeruginosa* during chlorination.^18^ However, there is limited information regarding the effect of Fe(vi) on the characterization and DBP formation of IOM and EOM.

Bromide (Br^−^) is detected in surface water in the range from 10 to 1000 μg L^−1^ as an important parameter for the formation of brominated DBPs.^19^ During chlorination, Br^−^ could be oxidized to form hypobromous (HOBr and OBr^−^) products, which had stronger oxidation ability to react with AOM to form brominated DBPs.^20^ A few studies have reported THM and HAA formation during chlorination at initial Br^−^ concentrations of 50 and 100 μg L^−1^.^21,22^ Besides, a previous study confirmed that Fe(vi) oxidation with a high concentration of Br^−^ could form a little amount of brominated DBPs.^23^ Brominated DBPs are cytotoxic and genotoxic to Chinese hamster ovary (CHO) cells,^24^ which made it essential to consider the effect of bromide on Fe(vi) pre-oxidation with subsequent chlorination.

EOM and IOM used in this research were derived from *Chlorella* sp. (green algae) and *P. limnetica* (blue-green algae), which were main algal species appearing in inland lakes. The goals of this study are as follows: (1) to characterize the effect of Fe(vi) oxidation on IOM and EOM; (2) to evaluate the impacts of DBP formation (4 THMs, 5 HANs, 2 HKs, chloral hydrate (CH) and nitrotrichloromethane (TCNM)) from IOM and EOM with pre-oxidation of Fe(vi) and subsequent chlorination; and (3) to investigate the effect of Br^−^ concentrations on the formation of brominated DBPs.

## 2. Materials and methods

### 2.1 Chemicals and regents

All chemical regents purchased were of analytical grade. Sodium hypochlorite (NaOCl, 5%) was obtained from Aladdin (Shanghai, China), which was diluted to 1000 mg L^−1^ (the concentration of Cl_2_) and used as a stock solution of free chlorine. Potassium ferrate Fe(vi) (K_2_FeO_4_) was obtained from the laboratory with high purity (>92%) based on the oxidation of ferric nitrate with hypochlorite.^25^ Fe(vi) stock solutions with an absorption coefficient of 1150 M^−1^ s^−1^ at pH 9.0 were freshly prepared and used within 30 min.^26^ The standards of 4 THMs, 5 HANs, 2 HKs, CH and TCNM (gas chromatographic grade) were obtained from Aladdin. Methyl *tert*-butyl ether (MTBE) (gas chromatographic grade) was purchased from Sigma-Aldrich (USA). Other reagents such as NaOH (≥98%) and H_2_SO_4_ were purchased from Sinopharm Chemical Reagent Co., Ltd (Shanghai, China). All stock solutions were prepared using ultra-pure water (Heal Force ultra-pure system, Millipore) with resistivity ≥18.2 MΩ cm.

*Chlorella* sp. (FACHB-25) and *P. limnetica* (FACHB-1277) were purchased from the Institute of Hydrobiology, Chinese Academy of Sciences (Wuhan, China), and cultured in BG11 media in a light incubator (12 h darkness and 12 h light) at 25 °C. IOM and EOM solutions were separated from algal solutions when the algal cells were in a stable growth phase. *Chlorella* sp. and *P. limnetica* solutions were centrifuged at 6000 rpm for 10 min, followed by two additional cycles of centrifugation and supernatant removal. The obtained supernatants were filtered using 0.45 μm GF/F membranes to obtain EOM. The deposited algal cells were re-suspended in Milli-Q water (18.2 MΩ cm) for freeze–thawing for three cycles, and then centrifuged at 10 000 rpm for 6 min before filtration through 0.45 μm GF/F membranes to obtain IOM.

### 2.2 Experimental procedures

Potassium ferrate solutions were prepared freshly just before Fe(vi) oxidation. The IOM and EOM solutions of *Chlorella* sp. and *P. limnetica* were placed in a thermostat water bath (100 mL) with the initial dissolved organic carbon (DOC) of 5 mg L^−1^ (pH was controlled at 7.0 with 0.2 M phosphate buffer). Fe(vi) was added to obtain the desired concentrations (0, 8 and 16 mg L^−1^). After stirring for 20 minutes (200 rpm), the solutions were divided into two subsamples. The first sample (20 mL) was collected and quenched with ascorbic acid, and then subjected to excitation emission matrix (EEM) analysis within 20 min. The second sample (60 mL) was used for subsequent chlorination experiments.

The aforementioned Fe(vi)-oxidized samples (60 mL) were prepared for chlorination. The chlorination experiments were carried out using 60 mL amber bottles at 25 °C. Before chlorination, the pH was adjusted to 7.0, the solution of sodium hypochlorite (1000 mg L^−1^) was then added to the desired ratio of initial free chlorine and DOC (6 : 1), and the pH was again adjusted back to 7.0. The chlorination process occurred in the dark at 25 °C for 72 h. The reaction was quenched after 72 h by adding 0.1 mL Na_2_S_2_O_3_ solution (200 mg L^−1^). Following this, 30 mL samples from 60 mL amber bottles were collected for DBP analysis. Besides the Fe(vi) doses, other various factors were also investigated, including chlorination pH (6.0, 7.0, 8.0, 9.0 and 10.0), ratios of initial free chlorine and DOC (3 : 1, 6 : 1, 9 : 1 and 12 : 1) and bromide concentrations (0, 0.1, 0.2 and 0.4 mg L^−1^). All the experiments were conducted with the IOM and EOM at an initial DOC of 5.0 mg L^−1^, and the chlorination time was fixed as 72 h.

### 2.3 Analytical methods

The procedure of liquid–liquid extraction (LLE) is described as follows: (1) a 30 mL water sample was adjusted to a pH of 5.0; (2) 2 mL MTBE and 8 g anhydrous sodium sulfate were added to the extract for better stratification; (3) the sample was shaken in a mechanical shaker for 5 min and then allowed to stand for 30 min; and (4) 1 mL organic solution was taken from the upper layer for DBP analysis.^27^ The quenched sample was subjected to the measurements of volatile DBPs (4 THMs, 5 HANs, 2 HKs, CH and TCNM) after LLE. The separation and identification of DBPs were achieved by gas chromatography using an electron capture detector (GC-ECD, Thermo Fisher), equipped with an Agilent HP-5 capillary column (30 m × 0.25 mm × 0.25 μm, Agilent, Santa Clara, CA, U.S.). The cycles of temperature are as follows: (1) 35 °C for 10 min; (2) heated to 80 °C at 10 °C min^−1^; and (3) heated to 150 °C at 20 °C min^−1^.^28^ The flow rate was 1 mL min^−1^, and the temperatures of the headspace injection and ECD detector were 210 °C and 290 °C, respectively.

DOC and dissolved organic nitrogen (DON) were measured using a TOC/TN analyzer (Shimadzu TOC-VCSH analyzer, Japan). UV absorbances at 254 nm (UV254), 253 nm (UV253) and 203 nm (UV203) were detected by UV spectrophotometry (HACH DR 5000). Then, the UV254 was divided by DOC values to obtain the specific UV absorbance (SUVA). The concentrations of Fe(vi) and free chlorine were also tested by UV spectrophotometry at detection wavelengths of 510 nm and 540 nm, respectively. The EEM measurement was used for characterizing IOM and EOM (Hitachi F-4600 fluorescence, Japan). The excitation emission (Em) was set from 220 nm to 400 nm with an increment of 2 nm, and the excitation (Ex) wavelength was set from 280 nm to 500 nm with an increment of 5 nm. The Em and Ex of aromatic proteins (I + II) were 220–250 nm and 280–380 nm, the Em and Ex of fulvic acid-like products (III) were 220–250 nm and 380–500 nm, the Em and Ex of soluble microbial-like products (SMP) (IV) were 250–280 nm and 280–380 nm, and the Em and Ex of humic acid-like products (V) were 250–400 nm and 380–500 nm.

## 3. Results and discussion

### 3.1 Characterization of IOM and EOM

The basic parameters (UV254, DON, SUVA and UV253/UV203) of EOM and IOM of *Chlorella* sp. and *P. limnetica* are summarized in Table 1. The UV254 and SUVA values of IOM were higher than that of EOM, indicating more aromaticity in IOM than in EOM. However, the ratio of UV253/UV203 of IOM was lower than that of EOM because the aromatic moisture of IOM was highly activated, and EOM had a higher substitution tendency of activated aromatic rings.^29,30^ These results were consistent with the results of previous studies, which showed that EOM and IOM of *Chlorella* sp. had a relatively low SUVA value (<2 L mg^−1^ m^−1^).^27^ Compared with *Chlorella* sp., EOM of *P. limnetica* had lower SUVA values due to the abundance of protein-like materials. Korshin *et al.* reported that the UV253/UV203 value and THM formation potential had a strong linear correlation,^31^ which indicated that IOM was likely a higher yielding precursor of THMs than EOM. Furthermore, IOM had more dissolved organic nitrogen than EOM, and hence, the ratio of DOC/DON in EOM was higher than that in IOM. The lower DON value in EOM than in IOM was attributed to the dominance of nitrogen-rich organic compounds (*i.e.* proteins and amino acids),^32^ which indicated that IOM had more potential to form N-DBPs. In addition, the DOC, UV254, DON and SUVA of the algal solutions decreased after Fe(vi) pre-oxidation. Fe(vi) could destroy the aromatic structures and oxidize macromolecular matters into small molecular matters.^33^ This result indicated that Fe(vi) pre-oxidation could remove organic matters to decrease the DBP formation potential in the following chlorination experiment.

**Table 1:** Basic properties of IOM and EOM with or without Fe(vi) pre-oxidation^a^

Parameter		DOC (mg L^−1^)	UV254 (cm^−1^)	DON (mg L^−1^)	UV_253_/UV_203_	DOC/DON	SUVA (L mg^−1^ m^−1^)
*Chlorella* sp.	IOM	5.0	0.060	0.960	0.019	5.21	1.20
	EOM	5.0	0.056	0.621	0.015	8.05	1.12
*P. limnetica*	IOM	5.0	0.061	1.385	0.071	3.61	1.22
	EOM	5.0	0.042	0.837	0.015	5.97	0.84
*Chlorella* sp. with Fe(vi)	IOM	4.1	0.051	0.898	—	5.57	1.02
	EOM	4.4	0.049	0.583	—	8.58	0.98
*P. limnetica* with Fe(vi)	IOM	3.8	0.048	1.310	—	3.82	0.96
	EOM	4.0	0.037	0.802	—	6.23	0.74

a pH was adjusted to 7.0, Fe(vi) was added to 8.0 mg L^−1^ for 20 min, *n* = 2.

**Fig. 1:**
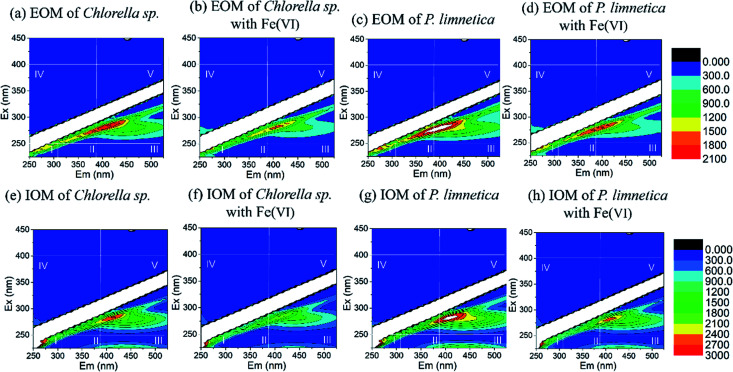
(a–d) Fluorescence EEMs of EOM from *Chlorella* sp. and *P. limnetica* with or without Fe(vi) oxidation. (e–h) Fluorescence EEMs of IOM from *Chlorella* sp. and *P. limnetica* with or without Fe(vi) oxidation. (Experimental conditions: initial DOC of IOM and EOM was 5 mg L^−1^, Fe(vi) dose was 8 mg L^−1^, oxidation time was 20 min, pH was 7.0).

Fig. 1 depicts the fluorescence EEM spectra of IOM and EOM in *Chlorella* sp. and *P. limnetica* with or without Fe(vi) oxidation. It could be observed that IOM and EOM solutions had the same highest peak at the SMP-like region (region IV), which indicated that IOM and EOM solutions had similar characteristics. However, EOM solutions of *P. limnetica* had two main peaks at SMP-like region (region IV) and humic acid-like region (region V), and IOM solutions showed three main peaks at SMP-like region (region IV), humic acid-like region (region V) and fulvic acid-like region (region III). The characteristics of IOM solutions of *P. limnetica* were more complex, dominated by nitrogen-rich, humic and fulvic acid-like compounds. Therefore, the IOM of *P. limnetica* had more potential to form HANs and TCNM.

Fig. 1b, d, f and h show the effect of Fe(vi) oxidation by the fluorescence EEM spectra of IOM and EOM in *Chlorella* sp. and *P. limnetica*. The normalized region-specific EEM volumes (*Φ*_i,n_) before and after Fe(vi) oxidation were calculated by the fluorescence regional integration (FRI) method.^34^ For *Chlorella* sp., the region IV of EOM and IOM was decreased by 7.3% and 5.5%, respectively. For *P. limnetica*, region IV of EOM and IOM was decreased by 12.6% and 9.7%, and region V of IOM was decreased by 5.1%. Fe(vi) oxidation changed the composition and structure of protein and microbial metabolites, resulting in a decrease in fluorescence.^35^ Compared with *Chlorella* sp., Fe(vi) had a greater impact on the EOM and IOM of *P. limnetica*.^36,37^ The changes in IOM and EOM (*e.g.* characteristics) by Fe(vi) influenced DBP formation in subsequent chlorination.

### 3.2 DBP formation in subsequent chlorination

#### 3.2.1 Effect of Fe(vi) dose

**Fig. 2:**
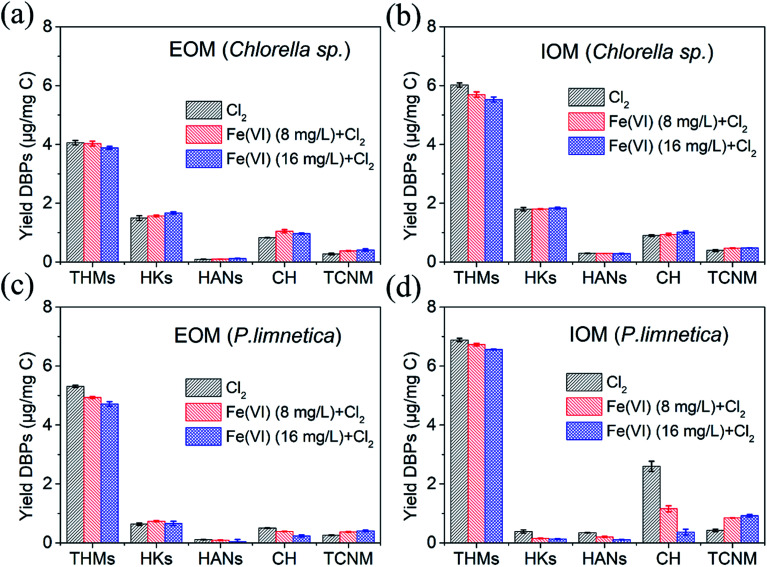
Formation of THMs, HANs, HKs, CH and TCNM during the chlorination of EOM and IOM after pre-oxidation with Fe(vi). (a) DBP formation from EOM of *Chlorella* sp., (b) DBP formation from IOM of *Chlorella* sp., (c) DBP formation from EOM of *P. limnetica*, and (d) DBP formation from IOM of *P. limnetica*. (Experimental conditions: Fe(vi) doses were 8 and 16 mg L^−1^, pre-oxidation time was 20 min, free chlorine (Cl_2_)/DOC = 6 : 1, pH = 7.0, temperature = 25 °C, chlorination time was 72 h).

The effect of Fe(vi) pre-oxidation (used in oxidation for 20 min) on the formation of THMs, HKs, HANs, CH and TCNM during the chlorination of EOM and IOM from *Chlorella* sp. and *P. limnetica* was investigated and shown in Fig. 2. First, THM formation was the highest among the tested DBPs, followed by HKs, CH and TCNM, and the concentrations of HANs were lower than 0.2 μg mg^−1^ C^−1^. The higher formation of THMs in chlorination with IOM and EOM was consistent with the previous studies.^7,38^ Second, the DBP formation from four solutions with chlorination alone followed the order: IOM of *P. limnetica* > IOM of *Chlorella* sp. > EOM of *P. limnetica* > EOM of *Chlorella* sp. IOM and EOM from *P. limnetica* had more dissolved organic matter (Fig. 1) and higher nitrogenous DBP potential (compared DON values in Table 1) than that from *Chlorella* sp., respectively. Besides, a previous study has confirmed that IOM had higher concentrations of 16 amino acids than EOM per milligram of DOC.^6^ The DBPs from IOM were more than those from EOM both in *Chlorella* sp. and *P. limnetica*. With the same DOC of solutions, IOM solutions showed more SUVA and DON (Table 1), which indicated that IOM had more aromatic carbon and N-DBP formation potential than EOM. A previous study showed that the intracellular soluble protein was 50% nitrogen-enriched.^3^ Third, for *Chlorella* sp., all kinds of DBP formation from IOM were more than that from EOM, but for *P. limnetica*, the HK formation from IOM (0.39 μg mg^−1^ C^−1^) was less than that from EOM (0.64 μg mg^−1^ C^−1^).

Compared with the DBP formation with chlorination alone, the DBP formation of EOM solutions in *Chlorella* sp. presented no significant change with the increase in Fe(vi) dosages. IOM from *Chlorella* sp. showed a reduction of THMs (8.2%) after Fe(vi) oxidation at a dosage of 16 mg L^−1^. The results of this research indicated that Fe(vi) presented relatively low performance for IOM and EOM removal. Zhou *et al.* found that the AOM removal could reach 50% after the pre-oxidation of high Fe(vi) doses.^18^ The removal of IOM and EOM by Fe(vi) was only caused by oxidation, while the removal of AOM involved flocculation and oxidation. EOM and IOM of *P. limnetica* both exhibited reduction of THMs and CH after Fe(vi) oxidation. However, Fe(vi) oxidation increased HAN and TCNM formation of both EOM and IOM. Fe(vi) could react with electron-rich organic compounds such as amines and olefins to decrease the THM precursor, and oxidize the alcohol and amino acid groups in IOM and EOM to decrease the formation of CH.^39,40^ According to the fluorescence EEM spectra of IOM and EOM in Fig. 1, IOM and EOM solutions of *Chlorella* sp. had similar characteristics and contributed to the same regularity of DBP formation. However, IOM solutions of *P. limnetica* had more nitrogen-rich, and humic and fulvic acid-like compounds. The IOM of *P. limnetica* had more potential to form HAN and TCNM. Therefore, the pre-oxidation of Fe(vi) could inhibit the formation of most DBPs in subsequent chlorination.

#### 3.2.2 Effect of free chlorine dose

**Fig. 3:**
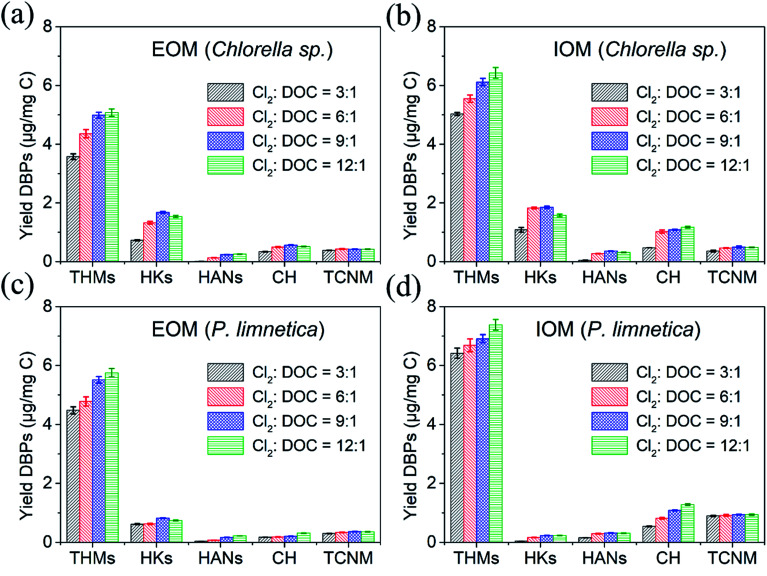
Effect of free chlorine on the formation of THMs, HANs, HKs, CH and TCNM during the chlorination of IOM and EOM after pre-oxidation. (a) DBP formation from EOM of *Chlorella* sp., (b) DBP formation from IOM of *Chlorella* sp., (c) DBP formation from EOM of *P. limnetica*, and (d) DBP formation from IOM of *P. limnetica* (experimental conditions: Fe(vi) doses were 8 mg L^−1^, pre-oxidation time was 20 min, pH = 7.0, temperature = 25 °C, chlorination time was 72 h).

Fig. 3 shows the effect of free chlorine doses on DBP formation from EOM and IOM after Fe(vi) oxidation. When the ratio of free chlorine to DOC increased from 3 : 1 to 12 : 1, THMs, HANs and CH monotonically increased, but HKs showed a trend of increasing first and then decreasing. THMs as the final DBPs could be stable in the presence of free chlorine. The concentrations of unstable DBPs (HANs, CH and HKs) were dependent on their formation and decomposition rates.^6^ When the free chlorine doses were enough, dichloroacetonitrile (DCAN) could be oxidized to trichloroacetonitrile (TCAN) (two kinds of HANs), and 1,1-dichloro-2-propanone (DCP) could be oxidized to 1,1,1-trichloro-2-propanone (TCP) (two kinds of HKs). The formation of HANs was higher than decomposition through oxidation or hydrolysis, attributed to the increase in free chlorine doses. However, the formation of HKs was lower than decomposition at high free chlorine doses. These results were consistent with the research results of Fang *et al.*^3^ The increasing free chlorine dose could decompose unstable DBPs. Besides, TCNM presented no significant changes with the increase in free chlorine dose. Chen *et al.* also found that TCNM concentration could not be changed greatly with the increase in chlorine or chloramine doses.^38^ This is because the limited nitrogen-containing organics in IOM and EOM have been oxidized to TCNM at low free chlorine doses, and more TCNM could not be generated with the increase in free chlorine doses. Therefore, only the formation potential of THMs depended on free chlorine doses.

#### 3.2.3 Effect of pH

**Fig. 4:**
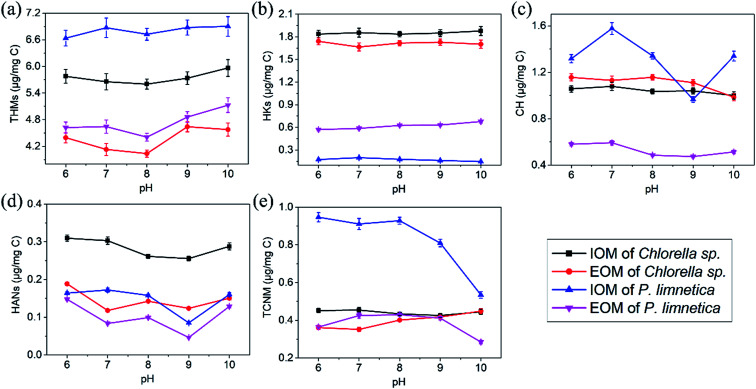
Effect of pH on the formation of THMs (a), HKs (b), CH (c), HANs (d) and TCNM (e) during the chlorination of IOM and EOM (*Chlorella* sp. and *P. limnetica*) after pre-oxidation. (Experimental conditions: Fe(vi) doses were 8 mg L^−1^, pre-oxidation time was 20 min, free chlorine (Cl_2_)/DOC = 6 : 1, temperature = 25 °C, chlorination time was 72 h).

As Fe(vi) was highly pH dependent, Fig. 4 shows the effect of pH on DBP formation during chlorination after Fe(vi) pre-oxidation. The results indicated that THMs had the lowest concentration at pH 8.0 in all four solutions (Fig. 4a). HKs and HANs showed slight changes with the increase in pH (Fig. 4b and d). CH from IOM and EOM of *Chlorella* sp. decreased with increase in pH from 6 to 10, and CH from *P. limnetica* had the lowest concentration at a pH value of 9.0 (Fig. 4c). In addition, TCNM from IOM of *Chlorella* sp. and *P. limnetica* showed a slight decrease when the pH value increased to 10, but TCNM from EOM showed a slight increase with the increase in pH from 6 to 9 (Fig. 4e).

Several reasons might contribute to these results. First, Fe(vi) could be decomposed rapidly when the pH was below 6.0 (the half-lives of Fe(vi) at pH 6 was 10^2^ s), and had a maximum stability at approximately pH 10 (the half-lives of Fe(vi) at pH 10 was more than 10^5^ s).^41^ Therefore, Fe(vi) was more chemically stable at high pH (pH > 9), and Fe(vi) had a significant oxidation potential at high pH values. Second, the decomposition of Fe(vi) generated Fe^3+^ to form Fe(OH)_3_ in an alkaline environment, which could remove partial DBP precursors with coagulation.^42^ The pH not only affected the coagulation and oxidation of ferrate, but also affected the reaction of free chlorine with precursors. Third, free chlorine had two existing forms of HOCl and OCl^−^ (p*K*_a_ = 7.5). When the pH increased from 6 to 10, more HOCl was translated to OCl^−^. As known, HOCl had higher oxidation potential with DBP precursors (*i.e.*, IOM and EOM) than OCl^−^.^43^ Finally, some unstable DBPs (HKs, HANs and CH) could be easily decomposed in an alkaline environment, such as *via* base-catalyzed hydrolysis.^44^ Based on the stability and oxidation of Fe(vi), pH was beneficial for reducing the formation of DBPs in the range of 8.0–9.0.

#### 3.2.4 Effect of bromide

**Fig. 5:**
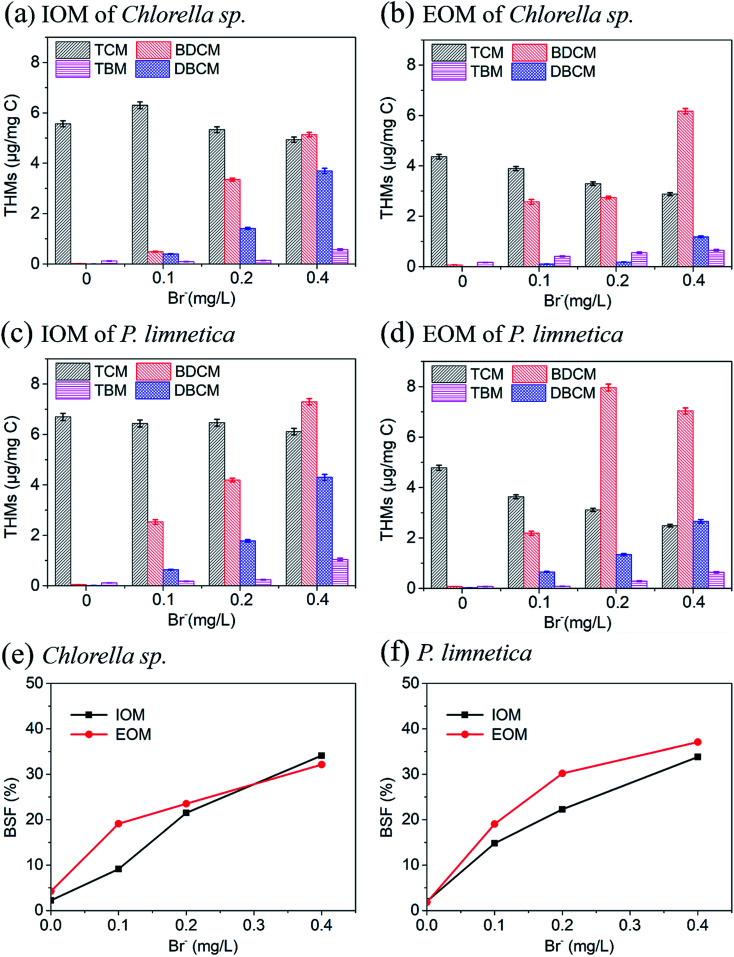
Effect of bromide on the formation of THMs, HANs, HKs, CH and TCNM during the chlorination of IOM and EOM after pre-oxidation. (a) DBP formation from EOM of *Chlorella* sp., (b) DBP formation from IOM of *Chlorella* sp., (c) DBP formation from EOM of *P. limnetica*, (d) DBP formation from IOM of *P. limnetica*, (e) the BSF of *Chlorella* sp., and (f) the BSF of *P. limnetica*. (Experimental conditions: Fe(vi) doses were 8 mg L^−1^, pre-oxidation time was 20 min, free chlorine (Cl_2_)/DOC = 6 : 1, temperature = 25 °C, chlorination time was 72 h).

The presence of bromine in the surface water primarily affected the presence of hypochlorous acid.^45^ The above-mentioned results confirmed that THMs were the main products formed during chlorination, more than any other DBP. Therefore, Fig. 5 shows the effect of Fe(vi) oxidation on brominated THM formation and bromine substitution factor (BSF) from IOM and EOM with the presence of different levels of bromide (0, 0.1, 0.2 and 0.4 mg L^−1^). The formation of THMs from IOM of *Chlorella* sp. (Fig. 5a) and *P. limnetica* (Fig. 5c) had a similar tendency. The formation of trichloromethane (TCM) decreased, and the formation of three brominated THMs (bromodichloromethane (BDCM), dibromochloromethane (DBCM) and tribromomethane (TBM)) increased with the increase in Br^−^ concentration. However, the formation of TCM decreased slowly, only decreased by 8.6% with the Br^−^ concentration increasing to 0.4 mg L^−1^. In addition, for EOM of *Chlorella* sp. (Fig. 5b) and *P. limnetica* (Fig. 5c), the formation of TCM decreased and the formation of BDCM increased significantly. There are two probable reasons for this phenomenon. The first reason is that HOCl/OCl^−^ could be oxidized to HOBr/OBr^−^ by Br^−^ in water. HOBr/OBr^−^ had more oxidizing efficiency with organic matter.^46^ A previous study also found that the reaction rates of HOBr and amines, olefins, aldehydes and alcohols were as high as 10^7^ M^−1^ s^−1^.^20^ The second reason is that the chlorinated DBPs could be further oxidized to brominated DBPs when the concentration of Br^−^ was sufficient.^47^ The ratio of Br^−^/Cl^−^ was the predominant parameter to determine the brominated DBPs formed during chlorination. Therefore, the formation of chlorinated DBPs was inhibited and brominated DBP was promoted, attributed to the increase in Br^−^ concentration.

BSF was introduced to determine the incorporation of bromide in DBP formation, which was calculated as the molar ratio of brominated DBPs to the total chlorinated DBPs and brominated DBPs in that class.^45^ It could be found that BSF-THMs of *Chlorella* sp. and *P. limnetica* both increased with the increase in Br^−^ concentration (Fig. 5e and f). When the concentration of Br-increased to 0.4 mg L^−1^, the BSF-THMs of IOM and EOM from *Chlorella* sp. increased by 31.9% and 21.8%, and the BSF-THMs of IOM and EOM from *P. limnetica* increased by 31.8% and 35.3%, respectively. In addition, for *Chlorella* sp., the BSF-THM value was lower in IOM than in EOM in the presence of low concentrations of bromine, and *vice versa* when the bromine concentration was 0.4 mg L^−1^. For *P. limnetica*, the BSF-THMs of EOM were always higher than IOM. Besides, compared with *Chlorella* sp., the value of BSF-THMs of *P. limnetica* was a little higher, which indicated that *P. limnetica* could produce more brominated DBPs with subsequent chlorination. Therefore, the increase in Br^−^ concentration in the algal solution mainly caused the increase in brominated DBP formation potential.

## 4. Conclusions

The effects of Fe(vi) on EOM and IOM of *Chlorella* sp. and *P. limnetica* have been completely studied, including characterization and DBP formation. The main conclusions drawn are as follows: (1) after Fe(vi) oxidation, soluble microbial-like products of EOM and IOM decreased, and humic acid-like products of IOM increased. (2) DBP formation on EOM and IOM of *Chlorella* sp. had the same regularity after Fe(vi) oxidation with subsequent chlorination. However, the IOM of *P. limnetica* had more potential to form HANs and TCNM than EOM. (3) THMs had the lowest concentration at pH 8.0 in all four solutions. HANs and HKs showed slight changes with the increase in pH. (4) All brominated DBPs increased, and chlorinated DBPs decreased with the increase in bromide concentration; (5) BSF of *Chlorella* sp. and *P. limnetica* both increased with the increase in Br^−^ concentration.

## Conflicts of interest

There are no conflicts of interest to declare.

## Supplementary Material
